# From Knowledge Graphs to Digital Twins: Perspectives on Modeling Patient Outcomes for Health Care Quality Assessment

**DOI:** 10.2196/81946

**Published:** 2026-03-31

**Authors:** Anna-Katharina Nitschke, Juan G Diaz Ochoa, Simone Neumaier, Markus Knott

**Affiliations:** 1 Department of Physics, Heidelberg University, Heidelberg, Germany; 2 PerMediQ, Pelargusstraße 2, Stuttgart, D-70180, Germany, 49 017650526323; 3 Institut für klinische und experimentelle Transfusionsmedizin, Tübingen, Germany; 4 Klinikum Stuttgart, Stuttgart Cancer Center, Stuttgart, Germany

**Keywords:** quality management, medicine, patient outcomes, machine learning, knowledge graphs, digital twins, human digital shadows

## Abstract

Medical applications of mathematical modeling, including machine learning models, knowledge graphs, and health digital twins, primarily involve the prediction of patient outcomes. This expert perspective examines how mathematical modeling can contribute to health care quality management. Definitions of procedures, patient outcomes, and quality metrics are provided with a quantitative focus. The emphasis is subsequently placed on 3 categories of patient-centered quality of care, namely, patient safety, procedure accuracy, and procedure efficacy, for which a conceptual and mathematical description is provided. Different levels of modeling tasks essential for managing patient-centered quality of care are identified. This article facilitates a deeper understanding of the topic by assigning relevant publications to these 3 quality categories. Focus is placed on the applicability of graph-based methods, including knowledge graphs and health digital twins, to improve quality management in health care. We have presented a clinical scenario and provided information on methodological limitations, future research directions, and practical implications.

## Introduction

In recent years, considerable efforts have been made to improve patient outcomes by standardizing medical care across health care systems for patients [1]. This has been achieved in particular through the creation and application of evidence-based medical guidelines [2]. Here, standardization (ie, the design of medical procedures via guidelines aimed at the systematic integration of scientific evidence) plays a relevant role. This is achieved by selecting the most appropriate health decisions required during the patient’s journey. Standardizing patient care is expected to improve cost and workflow efficiency, as well as resource allocation [3].

Many forms of medical interventions (eg, the selection of a therapy) can be seen as part of well-defined processes (ie, a series of actions taken to achieve an outcome) [4]. This concept intersects with the industry, where processes refer to a set of interconnected tasks that convert input into specific outputs. Therefore, modern medicine can be seen as a set of planned processes recommended by guidelines. This overlap has led to industrialization in medicine, with both positive (evidence-based treatments) and negative aspects (eg, dehumanization of medicine and focus on economic aspects) [3].

Furthermore, process-oriented health care focuses on process optimization and economic optimization instead of patient care (for a review of the industrialization of health care and its criticisms, see the chapter by Da Silva [5]). On the other hand, a patient-centered and process-oriented perspective can lead to positive aspects, such as breaking silos in clinical data, optimizing outcomes (ie, patient recovery after therapy), minimizing medical errors and expenses for diagnosis and treatment, and maximizing clinical outcomes [6]. In such cases, the balance between standardization and customization is a paramount problem [3].

Records containing medical interventions generated when a patient receives medical treatment (eg, electronic health records [EHRs]) are essentially defined as processes with different steps, including diagnosis, medical intervention (such as medication, surgery, etc), and tracking of a patient’s condition to prevent further health problems [7]. Thus, it is assumed that a patient’s process can be modeled in a similar way to the dynamics of a productive industrial process. Through the logical integration of different steps, both the well-being of the patient and the economic performance of the institution (eg, hospital) can be optimized.

Mathematical modeling can significantly improve the way medical processes are implemented and meet quality standards. However, medical data are often not used beyond direct patient care. The reasons for this are manifold and include a lack of digitalization, inadequate structured health data, stringent data protection laws, insufficient technical infrastructure, and limited personnel or financial capacities. Health care providers often use medical jargon and classification schemes that are country-specific and even center-specific (an exception is the use of the *International Classification of Diseases* [*ICD*] for disease classification), and information contained in the form of unstructured data is often not machine-readable.

In addition, health care practitioners (HCPs) are still not sufficiently trained in machine learning (ML) technologies. Moreover, IT specialists rarely understand clinical processes in health care facilities. In these dynamic times, it is crucial to bring different disciplines together if ML applications are to provide real benefits in everyday clinical practice [8]. Assuming that notions of causality can be identified in medicine and used to identify mechanisms and derive predictive models [9,10], ML applications can provide information about patient outcomes, as well as unexpected or undesired events, and support medical decision-making.

This article presents a novel perspective on modeling patient outcomes to improve health care quality assessment. The applicability of mathematical models (focusing on graph-based methods and health digital twins [HDTs]) is considered within a quantitative concept of health care quality management, considering 3 categories of patient-centered quality of care (PCQC), namely, patient safety, procedure accuracy, and procedure efficacy, thereby providing a consistent taxonomy. To date, this relevant topic has been explored only to a limited extent. This work aims to contribute to improving health and well-being (according to the UN sustainability goals, SDG3) [11]. We discuss the potential, limitations, and practical implications of the application of these technologies for modeling PCQC.

## Definition of Patient Outcomes for Quantitative Quality Assessment

### Overview

In medicine, it is generally difficult to define patient outcomes quantitatively (related to measurable information: exact metrics and observables captured by sensors) and qualitatively (related to nonmeasurable descriptions: categories) because patients are heterogeneous and complex organisms, and an overall assessment of their health status therefore remains a challenge.

Our goal is to identify these variables of interest and place them in a broader understanding of quality assessment, paving the way for mathematical modeling in this field. To this end, we aim to introduce a description of patient outcomes as a fundamental concept for evaluating quality indicators (QIs). These indicators are relevant for decision-making because they provide an estimate for which option holds the greatest desirability or value [12], that is, improvement of the quality of care (eg, improvement of patient well-being, avoidance of patient death, optimal use of resources, etc).

### Patient Outcomes

From a medical perspective, a patient outcome is defined in terms of what is meaningful and valuable to the individual patient [13]. However, this definition is very general and difficult to relate to a quantifiable metric. There is still a conceptual problem with the general definition of results in the medical context, such as patient-relevant outcomes (morbidity, mortality, and quality of life) and surrogate or biomarker outcomes [14]. These outcomes can differ significantly depending on the underlying disease. Liu et al [15] summarized the definition of patient outcomes related to clinical practice “as any change [within the patient’s health status] that results from health care [for which each profession] has developed outcome measures that focus on the standards, activities, and impact of its discipline.”

Thus, patient outcomes are manifold and range from PCQC [14] to institutional performance [16] and to patient-reported outcomes [17], which can be assessed from different data sources (for a comprehensive overview, see information provided by Busse et al [18]).

In this perspective article, we focus only on the description of PCQC (Table 1), for which a comprehensive review was published by Kersting et al [14]. We aim to provide a reference for the general concept, which is why we generally refer to medical procedures as follows: A *medical procedure* is the overarching phrasing for any type of observation, measurement, diagnosis, or treatment performed on the patient.

However, not all patient outcomes can be measured objectively, and only a limited number can be translated into clear, quantifiable metrics. In other words, a mathematical model can reflect only a fraction of the clinical reality.

**Table 1.** Subdivision of patient outcomes into 3 areas (patient-centered quality of care, institutional performance, and patient-reported outcomes).

Variable^a^	Patient-centered quality of care	Institutional performance	Patient-reported outcomes
Definition	Patient-centered QIs^b^ in health care [19] measured or captured by HCPs^c^ [14].	Process execution and resource consumption on an institutional statistical evaluation level [16].	Patient’s subjective evaluation of health experience. Report of the status of a patient’s health condition that comes directly from the patient, without interpretation of the patient’s response by a clinician or anyone else [17].
Exemplary categories	Medical evaluation of: Patient safety Procedure accuracy Procedure efficacy	Financial evaluation of: Patient waiting time Economic efficiency of medical procedures Productivity	Questionnaires evaluating: Symptoms Quality of life Health care experience

a For each area, a definition and a further subdivision into categories are given to enable a quantitative description of the different areas.

b QI: quality indicator.

c HCP: health care practitioner.

In the context of clinical trials, great efforts have been made to establish quantifiable patient outcomes. Clinical trials use predefined endpoints to measure patient outcomes. Typical endpoints are patient survival at a given time, incidence of clinical events (such as stroke), clinical performance measures, or patient-reported outcomes [20].

Considering the impact that EHRs have on the quality of care, we provide a definition of patient outcomes on the basis of changes registered in the health record and defined as events [21] (assuming that the data relevant for the definition of patient outcomes are machine-readable). We refer to *patient outcomes* as events registered in the health records. Such events can include changes in a patient’s health state or changes in medical procedures.

These outcomes enable health care providers and organizations to measure the impact of their services on patient well-being, identify key areas for improvement, and enhance the overall quality of care.

### Medical Guidelines

Medical guidelines are systematically developed statements that reflect the current state of medical knowledge. Medical experts prepare these guidelines in cooperation with professional associations. These evidence-based recommendations are based on clinical studies published in scientific literature and/or other existing evidence informed by expert experience and consensus.

The extracted information is then assessed and published by organizations, such as the World Health Organization, for international guidance or by country-specific professional societies (in Germany, the *Arbeitsgemeinschaft der Wissenschaftlichen Medizinischen Fachgesellschaften e. V*. [22]). Guidelines are not a static corpus and are continuously adapted and further developed on the basis of current clinical studies and scientific findings. They intend to support the decision-making of HCPs in order to enable appropriate care for certain health problems. Medical guidelines consist of complex workflows in mostly narrative form and can also contain multiple, equivalent treatment alternatives for the same disease unit. In general, comparing performed procedures with guidelines is challenging, especially considering that guideline recommendations should not be understood as rigid limits. In medical guidelines, a distinction is made between several grades of recommendations (“can” or “should”) on the basis of available evidence (see, for example, the AWMF register for prostate carcinoma [in German] [23]). Additionally, medical guidelines are country-specific and may differ across national states and regions.

### Information on QIs

To assess in part how guidelines are applied within an institution and to improve the quality of care, several QIs have been developed by different institutions. These QIs serve as internal quality management tools for medical institutions, enable benchmarking with other institutions, and aim to improve the quality of care for patients [24]. In Germany and Switzerland, routine billing data from hospitals are used for this purpose, if a health care facility decides to participate voluntarily in such initiatives [25]. In Germany, QIs for cancer treatment are defined annually by the German Cancer Society (*Deutsche Krebsgesellschaft* [DKG]). Hospitals that meet the criteria defined by the DKG are eligible to receive DKG certification. The outcomes of cancer patients treated in these DKG-certified centers are superior in terms of patient survival [26]. However, these QIs are assessed retrospectively on an institutional basis and cover aspects of the application of medical guidelines.

### Quality Metrics for PCQC

Mathematical modeling is used to represent a real-world phenomenon by capturing and analyzing the most significant relationships between different observables through formalized relationships between mathematical parameters. For the quantitative description of patient outcomes, they need to be described by numerically or categorically measurable parameters. These parameters capture various relevant aspects of the overall performance of patient-centered care quality. These numerical and categorical parameters are referred to as quality metrics (QMs) because they enable an evaluation of patient care. There is a hierarchical ordering that assigns each QI to several QMs, which might be defined in a disease- or context-specific way, to place them into the perspective of mathematical modeling. We refer to *QM* as a quantitative measure of QIs that relates to adherence to medical guidelines (procedural accuracy); QM also refers to patient-centered care (patient safety and effectiveness of the procedure).

## Information on PCQC

### Overview

We present an overview of the broad concept of PCQC. According to the classification provided in Table 1, we define in detail the 3 basic categories used for the further discussion of health care quality assessment: patient safety, procedure accuracy, and procedure efficacy.

For PCQC, there is a strong reference to medical guidelines and appropriate clinical practice. Owing to the increasing complexity of medical knowledge reflected within medical guidelines, it is becoming increasingly difficult for health care professionals to keep track of changes. The application of mathematical modeling is intended to help health care professionals comply with the guidelines (ie, a sequence of recommended procedures and support for medical decision-making). Medical practitioners select their decisions on the basis of the probability of initiating a change in the patient’s health state (assuming that physicians are either using heuristics or making rational decisions on the basis of available information [27]). Use cases for the application of mathematical modeling for PCQC are presented in Multimedia Appendix 1.

**Figure 1.**
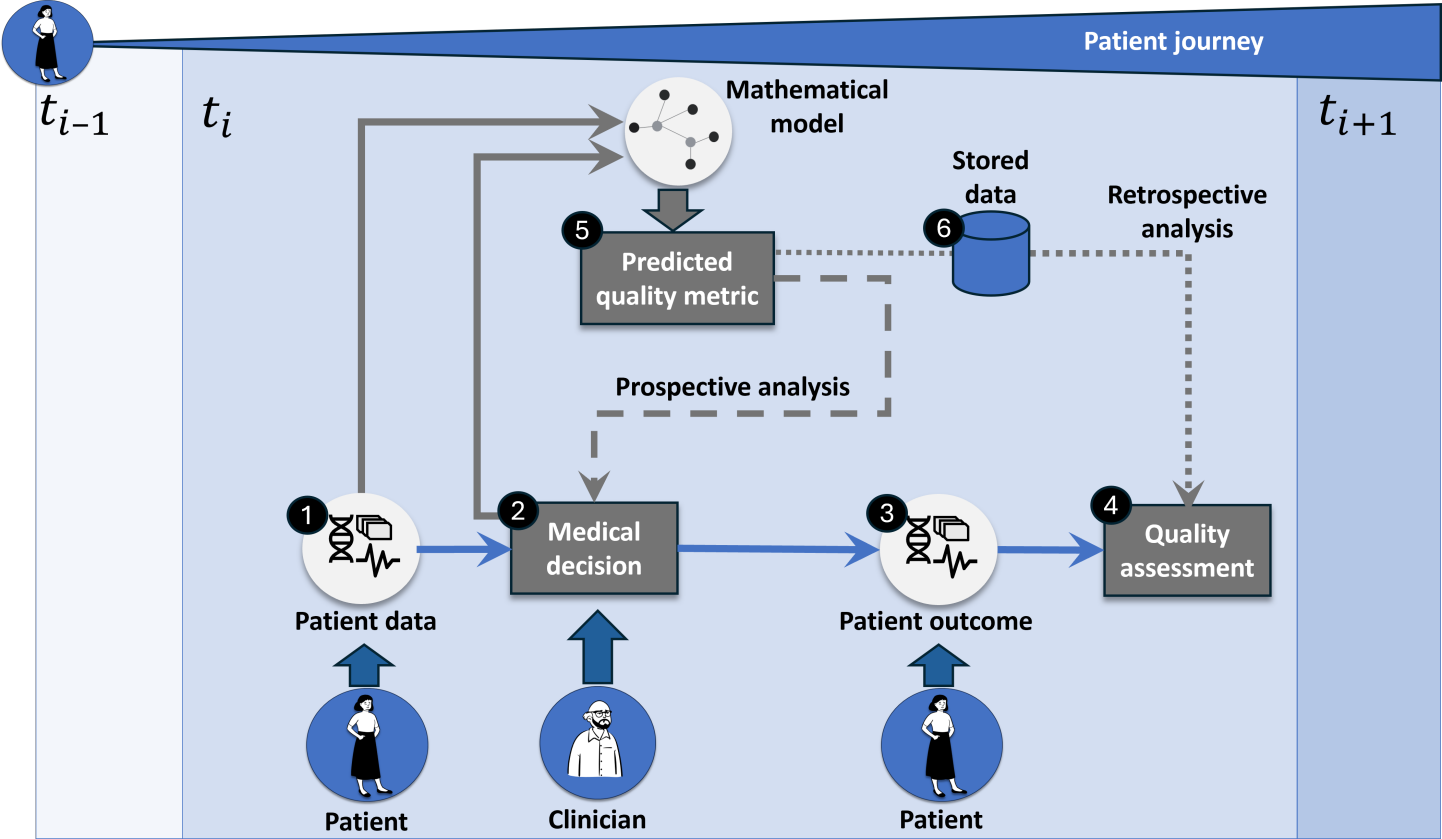
Visualization of key parameters for health care quality management and mathematical model integration throughout the patient’s journey over time. The predicted quality metric of the mathematical model can be implemented in 2 ways: prospective (dashed line) or retrospective (dotted line). It includes the following elements: (1) data collection from the patient, (2) medical decision made by a clinician, (3) patient outcome after the procedure, (4) quality assessment, (5) quality metric prediction from the mathematical model, and (6) data storage. This can analogously be performed at the population level (patient-cohort predictions) instead of at the patient level (patient-centered predictions).

Mathematical modeling can be included for predicting QMs to enhance health care quality. In this context, the patient’s journey can be understood as a complex chain of procedures that can be logically interconnected. Therefore, it is imperative to look at the whole patient journey as a process in which each procedure is just a point in time (*t*_*i*−1_, *t*_*i*+1_). As illustrated in Figure 1, the general way in which health care quality is assessed can be described as follows: collecting data from a patient (element 1), which a clinician uses to make a medical decision regarding the patient’s further procedure (element 2) and whose impact is measured after the procedure is carried out by looking at the patient outcome (element 3), which allows assessment of the quality of the health care provided (element 4). One can distinguish between two methods of implementation: (1) use the prediction in a prospective way (dashed arrow; meaning that before the medical decision is made, the clinician receives feedback from the model and can adjust the decision, leading to potentially different outcomes and improved health care quality) and (2) use a retrospective inclusion (dotted arrow), allowing an evaluation of the observed patient outcome and an assessment of the quality of care.

Another distinguishing feature of mathematical modeling is whether the model works for individual patients or for patient cohorts. Hence, for modeling the QM, we can consider the following representation levels:

1. Patient-cohort predictions for quality management of patient populations: It involves statistical modeling of the patient population for controlling domestic and global medical practices. At this level, mathematical models describe an “average and black box virtual patient” that tests or corroborates published and domestic guidelines.
2. Patient-centered predictions: It involves modeling of specific targets and the inclusion of personal information in gray (partial knowledge of model details) or white-box (full knowledge of model details) models to model “virtual individual patients” and evaluate published or domestic guidelines at an individual level. This implies that models should reflect and predict individual deviations from the expected results of the main patient cohort (see, for example, information provided by Peng et al [28]).

The patient journey can be represented as a chain of observed patient outcomes (for example, the main disease and corresponding codiseases) and possible medical decisions that lead again to distinct patient outcomes over time that are patient journey dependent. Medical guidelines specify possible medical decisions following patient outcomes. The quality of the health care received during the patient journey can be assessed by comparing different elements within this decision-tree structure. Three categories of PCQC can be distinguished: patient safety, procedure accuracy, and procedure efficacy. In the following sections, the 3 different categories of modeled patient outcomes are defined by describing their context to the patient journey in a qualitative and quantitative way, as well as identifying suitable QMs.

### Patient Safety

Providing an exact definition of patient safety is challenging, as this term remains under debate in the literature [29]. Patient safety (related to the taxonomy introduced in Table 1) can be measured by patient safety indicators, which refer mostly to the avoidance of accidental and unwanted events in health care. Patient safety is partially measured within health care facilities via predefined inpatient QIs. Patient safety indicators and inpatient QIs were developed by the Agency for Healthcare Research and Quality (AHRQ [30]), and together, they provide a comprehensive picture of QIs for patient safety. These QIs are mostly determined from the billing data of the hospitals and the medical documentation data contained therein (*ICD* codes for diagnoses and International Classification of Health Interventions [ICHI] codes [31,32] for medical procedures, although each country can have its own classification schema). Possible examples of QIs that reflect patient safety can be extracted from the AHRQ [30], such as complication-related indicators, procedure-related errors, medical-related errors, and hospital system indicators.

There are a variety of possible patient outcomes, some of which are desirable and some of which are adverse (an adverse outcome is evaluated depending on the specific clinical context). Assuming that physicians are rational decision makers, the medical decision is an evaluation of the risk of previous and future outcomes (according to the classic *Theory of Rational Decision Making*; see, for example, information provided previously [33]). A mathematical model for patient safety can provide support through checking (retrospective) or predicting (prospective) how likely the outcome of a medical decision will be an element of the set of adverse outcomes.

QMs can be related to the respective QIs for the quantitative representation of patient safety. Examples can be defined as follows: (1) Mortality rate and failure to rescue, which have emerged as important QMs [34]; (2) Emergency readmission to the hospital after a procedure among outpatients, which can happen, for instance, if prophylaxis has been forgotten or if safety guidelines have not been properly followed; (3) Number of reoperations or complications shortly after the operation, which might indicate a need for investigation within the facility; and (4) Admission to intensive care units (ICUs) and length of stay (LOS) in the ICU among inpatients (different from the intermediate medical care unit).

Table 2 depicts the relationships of the example QMs to the respective QIs, the patient status, the data type, and the QM type. The “Combination event with a time period” column indicates that the observed metric is provided in conjunction with a time parameter.

**Table 2.** QIs^a^ and QMs^b^, data type, correlation involving patient status, and combination event with a time period for patient safety.

Patient safety - QM	QI	QM kind	Data type	Correlation involving patient status	Combination event with a time period
Failure to rescue	Patient safety	Disease specific	Binary	ICHI^c^ with mortality	Yes (eg, within 30 days)
Hospital readmission	Hospital readmission	Context specific	Binary	—^d^	Yes (eg, within 30 days)
Number of reoperations or complications	Patient safety	Context specific	Numeric	—	Yes (eg, within 90 days or 1 year)
Admission to ICU^e^ and ICU-LOS^f^	Patient safety	Context specific	Binary	ICHI with critical care	Yes (eg, within 48‐72 hours)

a QI: quality indicator.

b QM: quality metric.

c ICHI: International Classification of Health Interventions.

d Not applicable.

e ICU: intensive care unit.

f ICU-LOS: length of stay in the intensive care unit.

### Procedure Accuracy

The purpose of medical guidelines is to reduce errors and disparities in medical care while supporting best practices and responsibilities in medicine on the basis of the current state of scientific knowledge. Following established medical guidelines increases the probability of achieving better clinical outcomes [35-37]. Therefore, an appropriate measure of PCQC (related to the taxonomy introduced in Table 1) is the adherence of HCPs to medical guidelines, including the correct and accurate application of a medical procedure, which we refer to as procedure accuracy. To date, there is no structured or automated assessment of whether recommended medical guidelines have been followed by HCPs for an individual patient.

For the inclusion of mathematical modeling for health care quality assessment, it is necessary to identify metrics that can be defined precisely (if possible, with binary or clear multilevel indicators). In most cases, these metrics cannot be defined universally and require disease-specific development. Thus, with respect to procedure accuracy, mathematical modeling can provide useful insights as follows:

- Anomaly detection and mathematical models can help discover common patterns in the relationship between diagnoses and medical procedures [38] or in the retrospective analysis of clinical narratives via natural language processing [39]. Such pattern recognition can be used to discover for which patient the pattern deviates from the guidelines and if such anomalies are an indication of a patient-centered procedure [40].
- Local guidelines (which are not merely deviations from national standards but adaptive tools shaped by real-world experience) can be created by hospitals or health care institutions. These include local adjustments to general guidelines when differences are identified, and the efficacy of alternative methods has been demonstrated [41].
- Patient-centered interpretation guidelines can be created, and the integration of additional data, such as physiological or genetic data, into models can provide recommendations for action that consider specific patient variations. Hence, models can enable the implementation of patient-centered quality management.

Clear results include checking whether the recommendations of a guideline for performing a standardized procedure or a set of procedures have been followed in an individual patient. This information can be retrieved from EHRs, laboratory information management systems, or even reimbursement data. In addition to compliance with a guideline, medical errors can also be reduced. Examples of QMs for procedure accuracy are as follows (Table 3):

- Detection of patient outliers: The average amount of hospitalization required by a medical procedure (LOS in hospital compared with the average) and the appearance of adverse events (number of infections, etc, compared with the average) can be assessed.
- Expected medical procedure: On the basis of the *ICD* diagnosis, specific ICHI procedures can be predicted, and deviations in the ICHI code from the prediction can be detected.
- Revision operation: The number of necessary additional surgical interventions after surgery (eg, due to complications of the initial surgical intervention) can be assessed.

While these metrics are easy to extract at the site level, they risk missing relevant contextual factors. It is desirable to integrate additional qualitative or clinical data points for a more holistic assessment. However, in clinical practice, simpler modeling techniques are used.

**Table 3.** QIs^a^ and QMs^b^, data type, correlation involving patient status, and combination event with a time period for procedure accuracy.

Procedure accuracy - QM	QI	QM kind	Data type^c^	Correlation involving patient status	Combination event with a time period
Detection of patient outliers	Accuracy of procedure application	Context specific	Binary	ICHI^d^ (patient) with ICHI (population)	No
Number of deviations of procedures with respect to guidelines (∆^e^ ICHI)	Accuracy in the application of medical guidelines	Context specific	Multilabel	ICHI (patient) with ICHI (population)	No
Revision of operations	Patient-specific management	Disease specific	Binary	—^f^	Yes (eg, within 90 days or 1 year)

a QI: quality indicator.

b QM: quality metric.

c Binary data correspond to 2 possible values (yes/no or 0/1).

d ICHI: International Classification of Health Interventions.

e The symbol ∆ refers to a difference with respect to a reference value.

f Not applicable.

### Procedure Efficacy

A general definition of efficacy has been provided by Lynch [42] (“efficacy is the capacity [of a medical intervention] to produce an effect”). For quality assessment, procedure efficacy (related to the taxonomy introduced in Table 1) refers to the optimal selection of procedures aiming at achieving the best possible patient outcome.

Retrospective models could compare the medical decisions chosen by HCPs with other possible choices, which may lead to better outcomes. Hence, procedure efficacy can also be considered for monitoring a decision (made under imperfect information) by observing the patient’s response or for making a prognosis and a subsequent decision under imperfect information. Owing to its nature, procedure efficacy requires the implementation of mechanisms and causal relationships where target/trial emulation is needed. This type of modeling contrasts with conventional predictive risk modeling tasks (which are applied, for instance, to evaluate patient safety and procedure accuracy).

The following are relevant QMs for procedure efficacy (Table 4):

- Biomarker levels: Predictions and projections of biomarker levels and their relationships with accepted levels to assess and predict a patient’s condition (acceptable [ie, considering parameters below a threshold] or deteriorating [ie, parameters above a threshold]) can lead to specific medical decisions. For example, a stable blood glucose range over time indicates the efficacy of diabetes treatment.
- Tumor-free survival: Time (in months or years) from complete tumor resection until tumor recurrence.
- Symptom risk score: This score quantifies, for example, the risk of an improvement or deterioration in a specific medical condition.
- Overall survival: Indicates the time (in months or years) from diagnosis until death from any cause.
- Pathologic complete remission: The proportion of patients showing no residual viable tumor cells on histopathological assessment after the completion of tumor therapy.

**Table 4.** QIs^a^ and QMs^b^, data type, correlation involving patient status, and combination event with a time period for procedure efficacy.

Procedure efficacy - QM	QI	QM kind	Data type^c^	Correlation involving patient status	Combination event with a time period
Biomarker levels	Surrogate for disease control	Disease specific	Time series	Biomarker with ICHI^d^	—^e^
Tumor-free survival	Tumor control	Disease/context specific	Binary	Biomarker/time	Yes (eg, after 1, 3, or 5 years)
Recurrence-free survival	Tumor control	Disease/context specific	Binary	Biomarker	Yes (eg, 12, 24, or 36 months)
Symptom risk scores	Satisfactory patient condition	Disease specific	Binary	—	Yes (eg, directly or within 6‐12 weeks after treatment)
Overall survival	Tumor control	Context specific	Binary	Biomarker	Yes (eg, after 1, 3, or 5 years)
Pathologic complete remission	Tumor control	Disease specific	Binary	Biomarker	Yes (eg, directly or within 6‐12 weeks after treatment)

a QI: quality indicator.

b QM: quality metric.

c Binary data correspond to 2 possible values (yes/no or 0/1).

d ICHI: International Classification of Health Interventions.

e Not applicable.

In legal or patient-communication contexts, terms such as “survivability” are typically used, which refer to an inherent capacity or probability of survival but are not considered valid clinical endpoints. In this study, we refer to well-defined outcome measures such as tumor-free survival.

## Mathematical Modeling of PCQC

### Overview

A mathematical representation of the patient’s condition to predict the outcome is essential for a precise evaluation of quality management. In summary, patient safety (Table 2) and procedure efficacy (Table 4) are related to clinical endpoints, whereas procedure accuracy (Table 3) is closely related to precision in the implementation of clinical guidelines and in reference to medical evidence. To avoid conceptual conflation in QMs, procedure accuracy is always defined with respect to documents (scientific literature or guidelines) containing medical or scientific evidence. Importantly, some events, such as patient outliers (Table 3), are estimated inside a time horizon and are mapped into binary states. Despite their convenience for mathematical modeling, there are relevant trade-offs, such as information loss or threshold choices, which can be problematic for the final validity of the models.

In the context of the 3 quality management categories, mathematical models need to perform distinct modeling tasks. First, the information coming from the patient needs to be stored and ordered in a data storage system (graph-based structures, such as knowledge graphs [KGs], can be used to encode patient data and information in a systematic manner). On the basis of the available data, single mathematical models (we focus on ML models) are trained to accomplish tasks that can either be classification tasks of the patient state or prediction tasks of the evolution of a parameter describing the patient response. The model predictions can be either prospective or retrospective.

We focus on KGs to integrate information from single events within the patient journey. By analyzing patterns from these KGs extracted via methods, such as graph neural networks (GNNs), the PCQC can be evaluated. For some QMs, it is necessary to consider the medical process in its specific context within the patient’s journey, which can be understood as a chain of events. This is an important aspect when assessing procedure efficacy, as it strongly depends on the previous and subsequent procedures. We present the concept of HDTs as a model integration framework, enabling a patient path representation.

The growing interest and relevance of mathematical modeling of patient outcomes are indicated in Table 5, which includes the number of published articles from the last 7 years (see Multimedia Appendix 2 for the methodology used to extract these data). However, the number of publications including digital twins (DTs) or GNNs is only a fraction of the total number of publications addressing the application of ML to predict patient outcomes (for a comprehensive review, see the article by Kline et al [43]).

**Table 5.** Publications in the last 7 years.

Variable^a^	Year, n						
	2019	2020	2021	2022	2023	2024	2025
Machine learning	899	1517	2289	2662	3192	4701	7146
Graph neural network	13	7	10	16	20	39	55
Digital twin	2	5	7	7	20	63	161

a Queries considering “patient outcomes” and “machine learning,” “graph neural network,” or “digital twin” (see Multimedia Appendix 2 for the method used to construct the table; the content was actualized on January 5, 2026).

### Graph-Based Methods for PCQC

With EHRs, extended research integrating different patient data for health care quality assessment is now possible. Regarding this development, Si et al [44] published a comprehensive survey about the use of current advances in patient representation based on data stored in EHRs.

In recent years, the application of linked knowledge in graphs has become relevant, in part because it allows holistic individualized representation, either by considering individual characteristics in an entire population [38] or by providing a patient-centered representation of the disease and the corresponding outcomes. Data representation as a graph is a method to leverage transparency in model construction. Graph models are also a way to map unstructured data, such as clinical narratives, into machine-readable data via large language models (LLMs). Additionally, graph models, including GNNs, have been considered to represent a way to close the gap between ML and symbolic reasoning [45], introducing the desired interpretability for ML methods.

**Figure 2.**
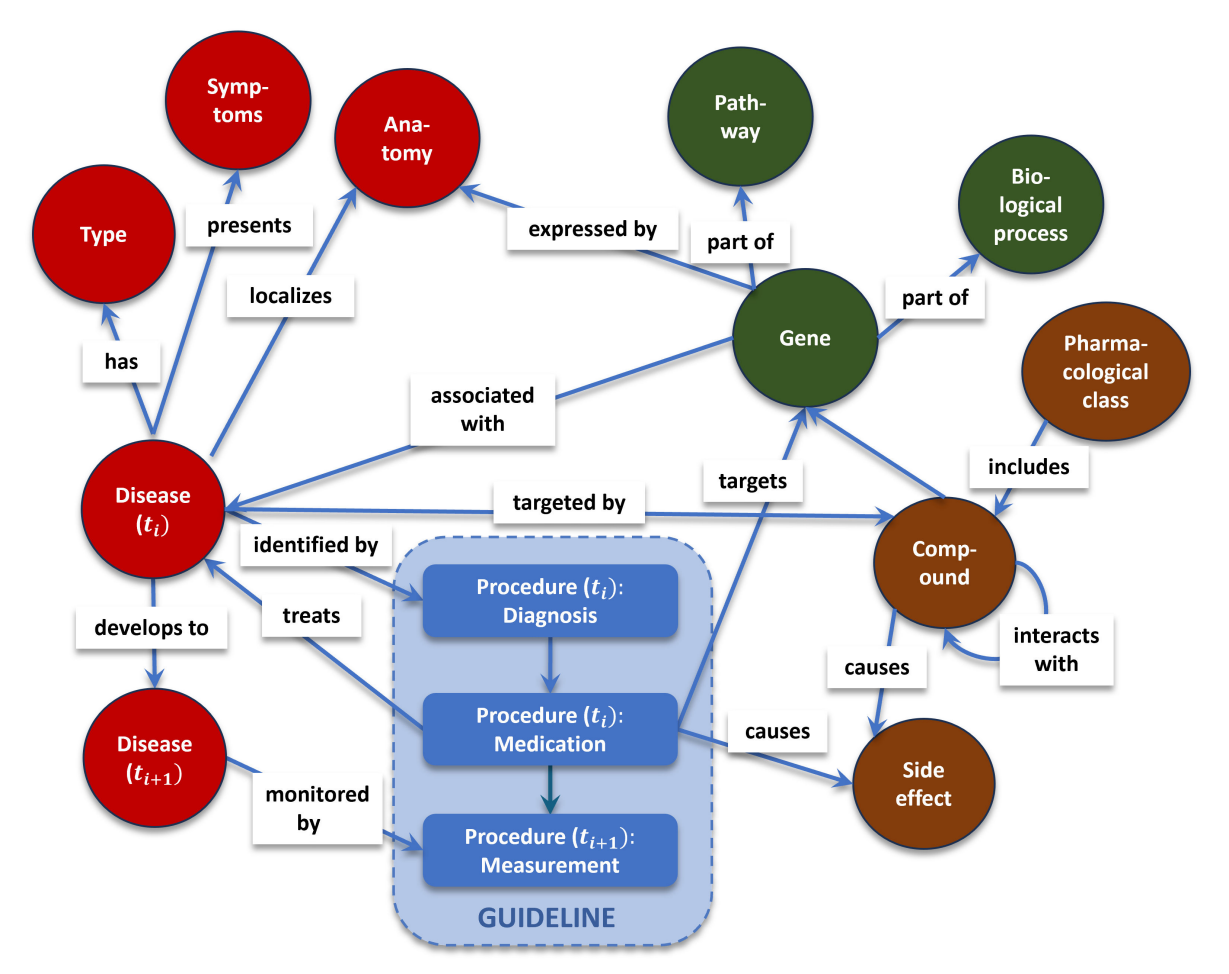
A simple health care knowledge graph inspired by the article of Abu-Salih et al [46]. The abstract graph shows how a disease is connected to its attributes (such as type, symptoms, and anatomy) and to compounds (and attributes such as side effects and pharmacological classes), and how it is associated with genes (and attributes such as pathways and biological processes). Interactions with the disease, which we refer to as medical procedures (such as diagnosis, medication, and measurement), can be added to the graph and can lead to the development of the disease over time (*t_i_*−*t_i_*_+1_).

The sample health care KG in Figure 2 can serve to represent the concept of graph models in health care: the health status of a patient can be modeled as the interlinking of various factors, such as the disease’s attributes (including type, symptoms, and anatomy), the patient’s genetics (including pathways and biological processes), the kind of drug compounds, and the characterization of side effects. Hence, graph models can function as holistic representations of a patient’s health status through the integration of different data sources into a single model.

In graph models, representation learning can be applied to convert multivariate time series and static features into nodes of the graph [47], for example, when multiple steps, such as various procedures in different time steps or disease stages, are converted into nodes in the entire graph.

Hence, starting from the disease stage at time point *t*_*i*_, procedures, such as diagnosis, can be performed, which introduces a new node into the KG. As clinical work is ideally performed within the frame of the guidelines, suitable medication will be proposed and applied. Any action taken through the performance of a medical procedure can lead to an evolution of the patient’s disease state, which, at a time point, can be monitored by the next procedure, again inducing new nodes in the KG. Relating medical procedures to specific patient characteristics (such as the genetic profile of the patient, side effects, etc) enables a holistic view, allowing guidelines to be tailored to the individual patient and to differ from institutional guidelines. Graphs are not limited to representing a single event and can represent a whole process (ie, the patient’s journey). This is useful for DT implementation, which will be discussed in the following section.

**Figure 3.**
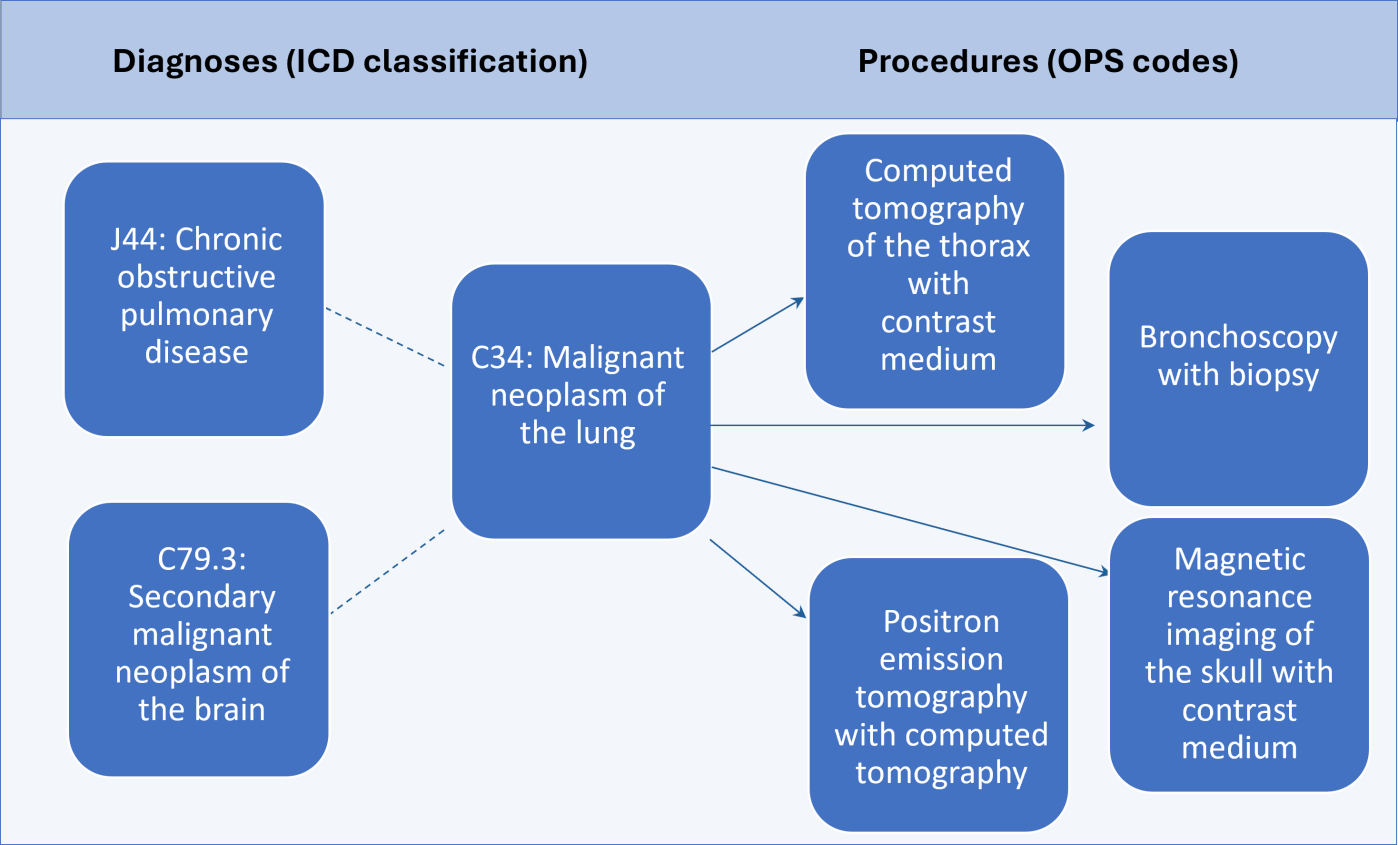
Exemplary knowledge graph related to disease diagnoses and medical procedures. In this example, we present the relationship involving a malignant neoplasm of the lung (C34). We also present typical codiagnoses of this disease on the left of the image (J44 and C79.3). On the right of the image, we present corresponding medical procedures that integrate the main diagnosis and codiagnoses (such as computed tomography, bronchoscopy, magnetic resonance imaging, and positron emission tomography). This example has been extracted from guidelines for lung cancer in oncology [48]. *ICD*: *International Classification of Diseases*; OPS: Operationen- und Prozedurenschlüssel.

In addition to patient-centered representations, graph models for disease-centered representations can also be constructed, as shown in Figure 3. These can be created from electronic patient records because hospital records contain rich information about the interrelationship of diagnoses (diagnoses and corresponding codiagnoses), the interrelationship of medical procedures (one medical intervention leads to another), and the interrelationship of diagnoses and procedures (due to the causal relationship between the diagnoses and the associated procedures). If a malignant neoplasm of the lungs (C34) is diagnosed, it is already known from earlier observations that it is often accompanied by chronic obstructive pulmonary disease (J44) and secondary malignant neoplasms of the brain (C79.3). Against the background of these relationships, 4 possible methods can be identified: computed tomography, bronchoscopy, magnetic resonance imaging, and positron emission tomography.

In Multimedia Appendix 3, we present an overview of the literature concerning the application of graph models for patient safety, procedure accuracy, and procedure efficacy.

### Limitations of Graph-Based Methods for PCQC

As the graph grows in size, querying and updating can become increasingly resource-intensive, potentially impacting the model’s performance. In addition, algorithms are often based on the message-passing paradigm, which consists of node-by-node iteration (where each node aggregates information from neighboring nodes), which has significant limitations for graphs consisting of highly connected nodes [49]. For this reason, graphs with explicit heterogeneous edge distributions pose a problem and are difficult to evaluate because of the excessive compression of exponentially growing information in fixed-size vectors [50]. Recently, methods based on a different approach than the node-centered approach, which is based on differential geometry, have shown promising results for overcoming graph bottlenecks [51].

KGs often have incomplete data. If the graph does not have all the relevant entities and relationships, the model may lack important information. Incorrect or outdated information can lead to erroneous insights and conclusions.

### HDTs and Health Digital Shadows for Modeling Procedure Efficacy

To model procedure efficacy, it is necessary to analyze the system’s (ie, patient’s) response to an external perturbation (eg, administration of a drug). It is crucial to gain deep insights into a patient’s condition to assess the efficacy of procedures. Here, we want to present how these modeling tasks can be accomplished by HDTs [52] and by health digital shadows (HDSs), specifically when using graph-based methods.

For modeling procedure efficacy, the targets are not only binary values indicating patient outcomes or medical procedures but also biomarkers recorded as time series. For example, it is currently not possible to predict in advance whether a patient will face an allergic reaction or intolerance to medication. However, the ability to predict such events is relevant, as these reactions can be life-threatening and lead to treatment delays or morbidity.

Another problem is that physicians lack the time to review all available medical records of a patient. In the case of complex diseases, such as cancer, with various genetic mutations that could change during the procedure, prior or regular modeling is needed. Procedure adaptations or medication adjustments can be made in cases of side effects or low procedure efficacy.

For example, genetic polymorphisms in drug metabolism are already known for some substances, which must be determined before the substance is administered (eg, dihydropyrimidine dehydrogenase testing before 5-fluorouracil administration). Possible drug interactions must also be considered if several diseases are present. Many patients take several medications, which can sometimes lead to unforeseeable interactions. For patients, these interactions are increasingly difficult to oversee and evaluate, and individual factors also play a role in tolerability. The combination of different target values, along with different time spans, makes the problem definition much more complex than for patient safety and procedure accuracy.

Hence, an algorithmic architecture capable of considering a wide range of patient information and potential interactions would help model patient responses to medical procedures, predict patient outcomes, and test different counterfactual scenarios (ie, estimating a patient’s outcome on the basis of a treatment regime that is distinct from the one that was actually followed and the observed outcome) [53,54]. Therefore, we model the integration of different procedures within a process. Technically speaking, this task represents the need for the integration of various models along a process (ie, patient’s journey).

For this modeling task, the concept of a DT, which originated from engineering sciences [55], can be adapted into a clinical context. The aim of a DT is to generate a digital representation of an existing object within a process or time evolution in a digital space. Its concept is designed to combine time-evolving information from real objects, such as data generated from the Internet of Things, and mathematical modeling in the digital space [56]. Therefore, this digital representation allows the adaptation or optimization of processes in a digital space before transformations or modifications of real processes are made. For example, by integrating different and disparate information sources, it is possible to create different models not only for industrial production but also for the solution of critical and strategic problems, such as pollution, by generating universal avatars [57]. In summary, the design of a DT has the following main characteristics [56]:

1. Enablement of bidirectional communication between the model and real assets.
2. Integration of different information sources, such as genetic information, comorbidities, medication history, medical history, and family history.
3. Integration of different models, such as mechanistic models (eg, physiologically based pharmacokinetic modeling [PBPK] models [58]) or inductive models (statistical models such as structured nested mean models or ML models), at one time point or for one procedure [53].
4. Coupling of individual procedure models within a process, for instance, in a patient journey.
5. Simulation of procedures over time and process.
6. Model improvement through learning from the digital cohort (sum of all DTs).
7. Compliance with context-specific requirements arising from the field of application (eg, clinical health service).

In the industry, objects can be fully digitalized (with a bidirectional flow between the object and the digital object), which is limited only by the current evidence and understanding of the underlying complexity of the physical or biochemical processes or materials that should be mimicked. However, in the medical context, patients are not only too biologically complex but also cannot be objectivized in a straightforward way [59]. For this reason, we prefer to refer to HDSs, even when the literature currently mainly uses the term HDTs [60].

Furthermore, the connection between KGs and HDSs represents a logical step in the way different and disparate information sources are connected to create descriptive and predictive models. Relevant patient information as well as medical guidelines (in the form of KGs) can be integrated into a full graph containing patient data (eg, genetic or socioeconomic data) and additional relevant information (eg, drug structure and drug interactions).

In its role as an information and model integrating method, an HDS is well-suited for the following [61]:

- Generic representations: The predicted value of a quantity of interest is within the expected range of the global population. The representations are on an abstract and qualitative scale.
- Population-specific representations: The predicted value of a quantity of interest is evaluated with respect to a patient cohort. Such representations are more precise, enabling quantitative predictions.
- Specific representations: The predicted value of a quantity of interest has high accuracy in relation to the expected value for a single patient. These models are used for personalized predictions.

The HDS concept is interesting for prospective analysis, that is, in the simulation and prediction of a patient’s health status and response to procedures. For prognostic purposes, it includes the ability to integrate all available patient information. Doctors treating patients can verify in advance the planned treatment regimen, such as medication or radiotherapy, on the patient’s digital representation.

The HDS model runs through all the interactions between the procedure and the patient. In the case of pharmacological treatment, medication interactions are simulated (eg, by integrating ML models for drug-drug interactions [62]). Then, an individual recommendation for the substance and the individual dosage and medication can be obtained by applying appropriate mathematical models (ie, ML). Additionally, if the treatment response is not sufficient, the HDS could propose additional procedures or further treatment strategies. Current databases for potential drug targets could also be considered.

**Figure 4.**
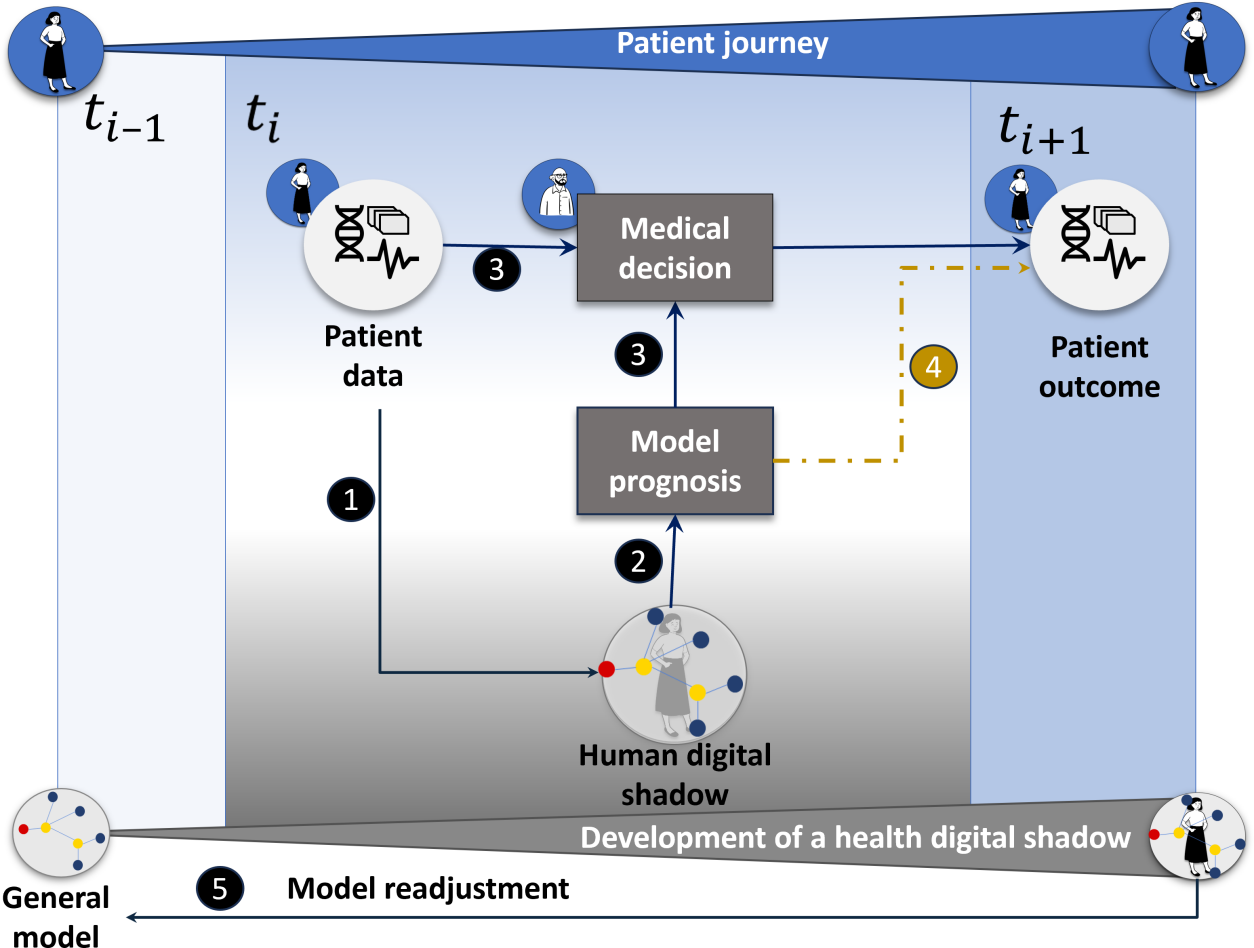
Representation of a health digital shadow (HDS) as an information system. The HDS integrates patient data into a generally derived mathematical model (1) and allows model prognosis (2). Thus, the model supports the work of medical personnel as additional information for medical decision-making (3), which leads to a patient outcome at the next time point. In this way, bidirectional communication between the HDS and the real world is enabled. The model is also able to simulate the patient’s journey, although the model’s prognosis can be used to directly estimate the patient’s outcome (4). The information gained by the individual human HDS can be used to readjust the general model constructed by the digital cohort (5).

Figure 4 shows how all these steps are included in a closed information loop. Bidirectional communication between the real world and the model is enabled. Therefore, the general model obtains patient-specific information at time point *t*_*i*_ (element 1). This enables the human HDS to make a prognosis (element 2), which can prospectively be used by the clinician to adapt the medical decision (element 3). Alternatively, model prediction can be used retrospectively to evaluate medical decisions by examining patient outcomes at time points *t*_*i*+1_ (element 4). As time evolves, the patient’s journey produces increasing patient-specific information, leading to better possible adaptation of the general model to a personalized human HDS. As the HDS cohort grows, model readjustments for the general model are performed after certain time periods (element 5).

Thus, HDSs could increase the safety of drug therapy and its efficacy by providing personalized recommendations based on all available information [63]. For example, a similar concept, which is based on a whole-body representation via PBPK models and the integration of multiagent systems representing hepatocyte metabolism, can be used for pharmacology, particularly for the representation of the avatar’s response to a medication [64,65]. This example demonstrates that through the analysis of the avatar’s response, it is possible to plan procedures and even consider individual patient characteristics (eg, personalized metabolite levels that depend on the patient’s genetics) that lead to personalized patient care.

This example implies that procedures can be tested on the DHS to forecast procedure success; therefore, the recommendation level of a procedure can be assessed before being applied to a real patient. Such an application is equivalent to the so-called dynamic HDT, where forecasts based on time series are performed [65].

Consequently, HDSs can be useful for modeling procedure efficacy, as in the following examples [66]:

- Human HDSs for inpatients:
  - We analyze which combination of medical procedures is most appropriate for patients depending on their socioeconomic context, environment, genetics, etc.
  - We analyze whether medical decisions will have an effective and desired outcome.
- Human HDSs for outpatients:
  - Help physicians recognize dangerous changes in a patient’s health state through the analysis of patient data that can be delivered in real time (wearables or machines, for instance, in nephrology).

**Figure 5.**
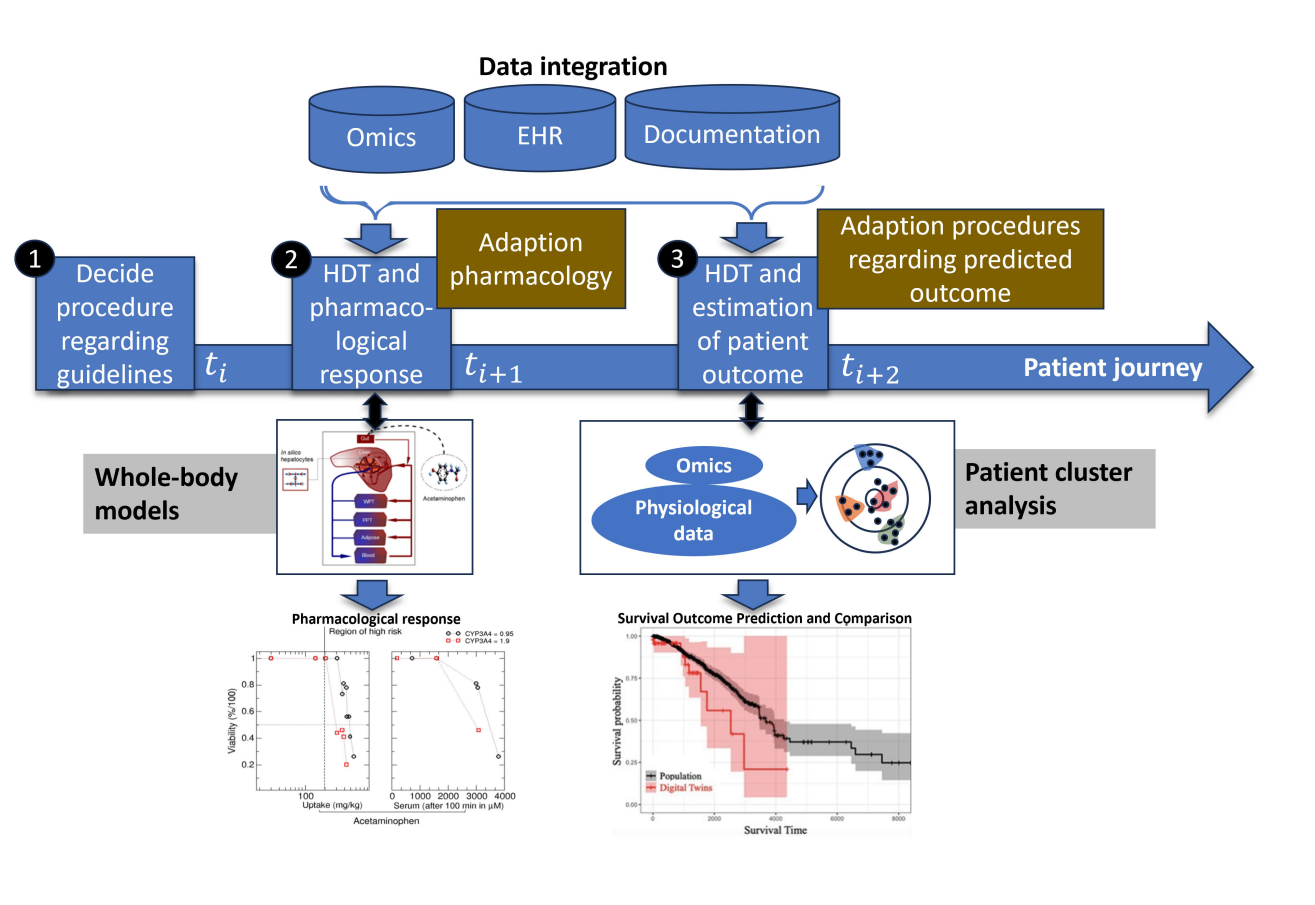
Schematic representation of a health digital shadow (HDS). An HDS is not a single model but a collection of different models to perform different predictions depending on the system’s parameters of interest. Such predictions can include the analysis and prediction of time series at different time points of the patient’s journey. In this image, we present a scheme of a patient’s journey: after making the first medical decision on the basis of clinical guidelines (1), different information sources are integrated to predict quality metrics in different time periods depending on the patient’s evolution (2). For example, the pharmacological response can be estimated via a whole-body model (eg, the physiologically based pharmacokinetic model [67]). Patient outcomes can be subsequently estimated via, for example, cluster analysis (3) [68]. EHR: electronic health record; HDT: health digital twin.

HDSs are increasingly being used for the estimation of the quality of patient care [55]. To this end, the prediction of patient outcomes is relevant, for example, in a personalized patient assessment to decide whether a procedure with high risk (death, side effects, patient burden, etc) is needed. This prevents the deterioration of a patient’s quality of life. Figure 5 shows an exemplary representation of a human HDS that integrates different data sources (such as omics, EHR, and documentation), which are measured in different time periods along the patient journey, into different models to predict patient outcomes.

As shown in Figure 5, after choosing the best possible medical procedure according to clinical guidelines, different information sources are integrated to predict QMs in different time periods depending on the patient’s evolution. For example, a whole-body (PBPK) model can be used to integrate patient-specific data within the HDT to predict a patient’s pharmacological response. Using pharmacological response prediction and patient data, another model can estimate patient outcomes (eg, through cluster analysis) for survival time prediction.

Chang et al presented a remarkable example of possible DHS realization through the creation of a pipeline for predicting patient outcomes, particularly survival heterogeneity, for patients with breast cancer [68,69]. In this implementation, 3 different data sources were integrated, where low-dimensional embeddings of clinical and molecular features enriched by an external annotation database were the main data sources. In this algorithm, a dimensional reduction method, Uniform Manifold Approximation and Projection, is implemented. This is a general-purpose manifold learning and dimension reduction algorithm to reduce the dimension of the data space. A dynamic HDS requires a pipeline that is accordingly adapted (ie, new data and additional models must be integrated and appropriately adapted according to other outcomes) [68].

Nitschke et al [68] presented a general and unspecialized design that is applicable for HDSs and is predictive, modular, evolving, informed, interpretable, and explainable. This design combines KGs and ensemble learning to represent the patient’s entire clinical journey and assist clinicians in their decision-making. Broad clinical application was shown by presenting 2 explicit cases: prostate cancer biopsy and glioma treatment decisions [68].

### Limitations of HDSs for Modeling Procedure Efficacy

HDSs often oversimplify real systems. These approximations can lead to discrepancies between the model and the actual system. The accuracy of the HDS depends on the quality and granularity of the data used. Since more than one model is coupled to generate a full HDS, different models trained on different data and granularities can lead to unbalanced representations. Finally, poor or sparse data can lead to unreliable models [70]. Notably, all these problems are exacerbated by issues related to the aggregation and integration of different types of data that occur in health care, along with the strict privacy policies that must be followed [71], a problem that may be alleviated with the introduction of EHRs [72].

Creating and maintaining an HDS can be computationally intensive, requiring significant resources for simulation and real-time data processing. Integrating an HDS with other systems, sensors, and data sources can be complex and may require sophisticated interfaces and protocols. Ensuring that the HDS reflects real-time changes in the physical system can be challenging, especially in systems with high dynamics [73].

An interesting option is the relation and validation of HDSs using in vitro methods, such as organ-on-a-chip. This option opens the possibility to compensate for potential defects in model predictions and identify their potential problems by validating and comparing results with advanced in vitro patient representations [74]. This, however, is still a challenge in the field of oncology and requires more research to obtain a correct representation of patient outcomes.

### Example of the Implementation of KGs and HDSs

The clinical scenario in Textbox 1 illustrates the integration of KGs and HDSs and demonstrates how our proposed framework evaluates all 3 QM categories while simultaneously assessing treatment safety in patients with comorbidities, ensuring guideline accuracy while allowing for personalized adaptation, and optimizing efficacy through predictive modeling of multiple therapeutic pathways for patients with non–small cell lung cancer. This case highlights how KG/HDS systems add value beyond static guidelines by dynamically integrating genomic data, resistance patterns, and real-world evidence to support complex clinical decision-making and establish prospective monitoring protocols.

Textbox 1. **Clinical scenario.**

A 64-year-old never-smoker with advanced lung adenocarcinoma undergoes comprehensive genomic profiling. Data: electronic health record showing diagnosis code C34.1 with brain metastases present; next-generation sequencing results showing EGFR L858R primary, T790M resistance mutation, and concurrent TP53; PD-L1 tumor proportion score showing a result of 2%; and baseline liver function test showing an alanine aminotransferase level of 45 U/L. Knowledge graph identifies treatment hierarchy according to National Comprehensive Cancer Network guidelines and maps resistance patterns to subsequent therapy options. Health digital shadow predicts the intracranial response rate with osimertinib (efficacy quality metric [QM]=0.70), hepatotoxicity probability (safety QM=0.18), and time to next progression (median 11.2 months). Accuracy assessment flags missing baseline brain magnetic resonance imaging according to the guidelines (accuracy QM=0.85). The system recommends osimertinib 80 mg daily with a central nervous system–specific imaging schedule, hepatic monitoring every 2 weeks initially, and liquid biopsy at 3 months for emerging resistance detection. The model also preidentifies next-line options based on likely resistance mechanisms, enabling proactive treatment planning.

## Issues and Future Research Directions

Our analysis revealed that patient safety, the accuracy of the procedure, and the effectiveness of the procedure are paramount. Using the present taxonomy, we can define the types of challenges and the type of ML implementation to be performed. However, this taxonomy is far from perfect. For example, distinguishing between a diagnostic procedure and a therapeutic procedure may require a more specialized and precise taxonomy. In addition, the metrics required to evaluate patient outcomes and estimate the QI may not always be perfectly calibrated [75], which can limit the applicability of such methods. In general, patient outcomes are still highly debated [76]. Despite this, we believe that it is important to keep any classification and taxonomy as simple as possible to facilitate the implementation of mathematical models.

We concluded that holistic quality management analysis (especially regarding procedure efficacy) requires the integration of several data sources. This is because there is no single decision (in decision support) or event (assignment of a diagnosis), but whole processes impact the patient’s health status. Quality management and assessment can be very challenging for model implementation since it requires the integration of a constant information flow, which can continuously change within the process. As a possible solution to address these issues, we have introduced the concept of the human HDS.

Guidelines serve as guidance to define and implement processes necessary for the management of patients’ health status. They are the result of clinical studies and are intended to help doctors make decisions. However, they contain complex workflows that should be evaluated individually. While a deviation from a guideline may be a potential problem in disease management for one patient because a step in the workflow has not been taken into account, which is a reduction in the quality of the hospital (ie, a decrease in procedural accuracy), for other patients, this deviation can be seen as a necessary adjustment needed for more personalized health care (ie, increasing the effectiveness of the procedure). In this context, especially in the evaluation of personalized practices, ML methods can influence the way guidelines are applied. Furthermore, we are cautious about full model–assisted personalization as well. Some critics have reported that data integration and target modeling have the potential to move away from real personalized patient centers and more human medicine [77]. In this case, individual modeling, which is embedded in a full modeling lifecycle, as shown in Multimedia Appendix 4, is just a way to quantitatively evaluate guidelines and their potential effects on the expected quality management of the disease at the patient level.

In this framework, graph representation is appealing, given that graph models are essentially an integration of data and information. Furthermore, when time is considered, it is possible to generate a digital representation of the patient (ie, an HDS). Furthermore, this concept has considerable potential for both inpatients and outpatients. This assumes that data generated by portable devices can track the patient’s condition at home. Furthermore, this concept can be coupled with digitally stored patient journeys (ie, the digital cohort) [78].

Currently, an HDS essentially involves the integration of different models within a process. We believe that in the future, the application of KGs will contribute to the rapid development of HDSs. For example, recent work has suggested that the combination of KGs and LNNs could be the key to the implementation of productive avatars in medicine [79].

Novel decentralized data storage concepts may help train and define accurate models with sufficient data volumes. For example, by integrating different and disparate information sources and real-world data built on the FAIR (findability, accessibility, interoperability, and reusability) principle [80], some of which are interconnected through the semantic web (according to the Worldwide Web 3.0 standards, for example, semantic web, such as Solid [81]), it is possible to create models not only for industrial production but also for the solution of critical and strategic problems by generating universal avatars [57]. Several recent initiatives are developing such ideas to implement DTs on the basis of semantic web principles (see, for instance, the EDITH-CSA project [61]). In such initiatives, it is remarkable to find not only the definition and exploration of models but also the definition of data standards required for data interoperability, most of which are based on Fast Healthcare Interoperability Resource (FHIR) standards.

HDSs face additional challenges. In the event of poor data safety standards, criminals could gain access to medical records and HDSs for blackmail or ransom demands. Companies, such as health insurance or disability insurance, might also demand the patient’s HDS prognosis before enrollment. Patients could be denied insurance, insurance rates could be increased, or existing insurance conditions could be adapted regularly on the basis of HDSs. This could ultimately lead to the selection of people who will not receive insurance at all or who will receive only reduced coverage. Institutions and employers could also demand an HDS model before hiring people in order to select healthy people.

Another relevant issue is data contamination by ML models. For example, if a machine catches early signs of sepsis and doctors treat it, this creates a “contaminated association” in the data. Furthermore, an intervention triggered by one model can quietly disrupt another, even if they are focused on entirely different outcomes [82]. This can be particularly critical for HDTs. A potential solution is to preserve clean data from datasets where predictive models are not used.

The level of mandatory treatment recommendations based on HDSs must be discussed and assessed. Patients’ wishes are crucial. What happens if the physician or patient deviates from an HDS’s recommendations? In this case, the insurance company may not cover the alternative treatments since the HDS does not predict them to be most effective. This question is related to the overall reliability of HDSs. In retrospect, the accuracy of such recommendations can be assessed (eg, by checking procedure efficacy and tolerability).

In summary, maintaining a healthy balance between the sense of euphoria and the concern for validity in the implementation of mathematical models is imperative. Graph models require clean labels, accurate graph structures, and sufficient initial node features; however, real-world graph data often have noise and sparse labels, whereas different datasets have distinct feature constructions [83]. Furthermore, DHSs often oversimplify the systems they represent, are challenged by in vivo conditions, and are difficult to maintain.

Recently, foundation large x models (LxMs) trained on large databases (eg, LLMs) have accelerated the implementation of most mathematical models while reducing development costs. However, these models are expensive and difficult to generalize (even LLMs have limited applicability). In such cases, a balance between LxMs (for instance, LLMs) and tailored models (for instance, BERT [Bidirectional Encoder Representations from Transformers]) can be the most suitable, cost-effective, and safe solution to all these challenges.

## Implications of the Study for Practice

The following steps and checks should help minimize some of the limitations of KGs applied to PCQC:

- Define a realistic system size that can be represented by KGs and maintain a critical view of the overscaling and size of KGs.
- Define aggregation functions to derive partial graph folding and compensate for incomplete node categories [51].
- Keep the graph updated with new knowledge and ensure consistency across different sources [84].
- Define appropriate data structures, such as Resource Description Frameworks, to make KGs interoperable.

Similarly, the following checklist can guide the correct implementation of HDSs applied to PCQC:

- Ensure data harmonization and minimization/access control of different data sources and digital records.
- Implement human-in-the-loop overriding for the correct interpretation of HDSs and avoidance of problems such as alert fatigue.
- Use drift detection/recertification and calibration standards (eg, using organ-on-a-chip) for safety in QM implementation on HDSs.
- Implement staged rollouts (eg, silent trials) before HDS activation.

Finally, the following are the key findings and conclusions derived throughout this article:

- Patient outcomes are not abstract quality terms since they can be formalized and linked to measurable clinical processes.
- Medical guidelines are essential but often underutilized in quality monitoring.
- Real-world clinical decisions are complex and require personalization.
- KGs and GNNs help bridge fragmented clinical data.
- HDSs offer a vision for prospective, patient-specific quality evaluation.
- Quality management should shift from static checklists to dynamic, data-driven guidance.

## Conclusion

The integration of KGs and DTs into health care holds transformative potential for improving clinical outcomes and the quality of care. However, this optimistic view is feasible only when the technologies are implemented in an ethical way, avoiding blind customer overconfidence in the delivered results. Furthermore, implementation requires accounting for the perspectives of stakeholders (as well as their own cultural environment and background): What is considered a desirable outcome from a patient-centered perspective (eg, symptom relief and preserved quality of life) may diverge from institutional or systemic goals such as shorter hospital stay or cost efficiency.

As such, mathematical models for health care quality must clearly define whether the primary focus lies in maximizing individual patient well-being, the operational performance of the health care system, or economic sustainability. These dimensions are not mutually exclusive, but the prioritization among them fundamentally shapes the modeling framework and interpretation of safety-related events. From this perspective, we also provide concrete checklists and recommendations for the correct deployment of models, for example, for the generation of KGs or the deployment of HDSs.

The combination of patient outcomes, procedures, and guidelines is not only interesting from a technical point of view but also extremely relevant for improving the quality of hospitals and health centers. The integration of guidelines implies the application of ML in different ways, from alerting and predicting critical conditions to the application of similar methods used in fraud detection to recognize potential deviations from guidelines.

The topic of quality management in medicine and the implementation of more detailed mathematical definitions are expected to become as relevant as diagnosis and image recognition are today, and we expect an increasing interest in further ML applications for quality management in the next few years.

## Supplementary material

10.2196/81946 Multimedia Appendix 1 Insight into the different aspects of the assessment of patient-centered quality of care through possible mathematical modeling applications.

10.2196/81946 Multimedia Appendix 2 Analysis of the available literature on PubMed.

10.2196/81946 Multimedia Appendix 3 Literature overview of the application of graph models for patient safety, procedure accuracy, and procedure efficacy.

10.2196/81946 Multimedia Appendix 4 Lifecycle of the data and models in relation to clinical guidelines.

## Abbreviations

AHRQ: Agency for Healthcare Research and Quality
BERT: Bidirectional Encoder Representations from Transformers
DKG: Deutsche Krebsgesellschaft (German Cancer Society)
DT: digital twin
EHR: electronic health record
GNN: graph neural network
HCP: health care practitioner
HDS: health digital shadow
HDT: health digital twin
*ICD*: *International Classification of Diseases*
ICHI: International Classification of Health Interventions
ICU: intensive care unit
KG: knowledge graph
LLM: large language model
LOS: length of stay
LxM: large x model
ML: machine learning
PBPK: physiologically based pharmacokinetic modeling
PCQC: patient-centered quality of care
QI: quality indicator
QM: quality metric
