# Supplementary material for: From Knowledge Graphs to Digital Twins: Perspectives on Modeling Patient Outcomes for Health Care Quality Assessment
Source: J Med Internet Res. 2026 Mar 31;28:e81946. doi: 10.2196/81946 (PMC13037766; doi:10.2196/81946)
Supplement: Multimedia Appendix 1 [file jmir-v28-e81946-s001.docx]

Appendix 1

Table A1-1 provides insight into the different aspects of the assessment of patient-centered quality of care through possible mathematical modeling applications in a prospective or retrospective way. Therefore, a **retrospective** approach involves collecting and analyzing data that have already been recorded, such as medical records or historical databases. In contrast, a **prospective** approach entails systematic collection of new data over time, often through direct observation, surveys or experiments, and model prediction.

Table A1-1 **Identified use cases for quality metrics following the taxonomy introduced in this article**

| Use Case | Description | Example of Potential Application of Mathematical Modeling |
| --- | --- | --- |
| Use Case 1: Patient Safety: *real time patient/biomarker tracking* | Real time information about the patient’s condition provided by measurements (e.g. by wearables), allows monitoring of prespecified deviations indicating a critical patient’s health state. | Software alerts to Health-Care Practitioners (**HCP**) immediately so that information will not get missed and direct actions can be taken (avoids information delay, bad documentation, or miscommunication). This can be for example implemented for real-time measurement of blood glucose increases or decreases in outpatients (however, such technologies still require appropriate implementations and cautious use in order to avoid overdiagnosis [1]) |
|  |  |  |
| Use Case 2: Procedure Accuracy: *retrospective analysis of QIs* | Control of the overall quality of a health center by observing the compliance to guidelines (audit in the application of guidelines) or whether local QM specifications are fulfilled or tracking of potential deviations in the healthcare process that might lower the quality of care (for instance adapting concepts from process mining in industry[2]) | A quality manager uses a mathematical model on retrospective data to track if certain complications after an operation have significantly increased. If this is the case, investigations in the affected department need to be initiated. |
| Use Case 3: Procedure Accuracy: *prospective* *reminder system (clinical decision support)* | Generated check lists/graphic visualization provides guidance to physicians by giving an overview of all possible procedures as well as a recommendation of the most suitable procedures regarding the medical guidelines. This is possible using a recommender system that filters all the possible procedures. | Depending on the patient's state, the mathematical model reminds HCPs for necessary procedures and checks if all previous procedures have been performed and otherwise reminds HCPs. |
|  |  |  |
| Use Case 4: Procedure Efficacy: prospective analysis for *deep insight* | Many patients have complex and long-term diseases and have been treated in different hospitals. It is time consuming for HCP to overlook all aspects of a patient’s journey. | A DT could provide a longitudinal overview of the patient’s medical journey and integrate all available data into the procedures. It could then offer suggestions for further procedures within the frame of the medical guidelines by generating a DT [3]. |

1. Segnan N, Ponti A. Artificial intelligence for breast cancer screening: breathtaking results and a word of caution. Lancet Oncol Elsevier; 2023 Aug 1;24(8):830–832. PMID:37541268

2. Rudnitckaia J, Venkatachalam HS, Essmann R, Hruška T, Colombo AW. Screening Process Mining and Value Stream Techniques on Industrial Manufacturing Processes: Process Modelling and Bottleneck Analysis. IEEE Access 2022;10:24203–24214. doi: 10.1109/ACCESS.2022.3152211

3. Katsoulakis E, Wang Q, Wu H, Shahriyari L, Fletcher R, Liu J, Achenie L, Liu H, Jackson P, Xiao Y, Syeda-Mahmood T, Tuli R, Deng J. Digital twins for health: a scoping review. Npj Digit Med Nature Publishing Group; 2024 Mar 22;7(1):77. doi: 10.1038/s41746-024-01073-0
