# Supplementary material for: From Knowledge Graphs to Digital Twins: Perspectives on Modeling Patient Outcomes for Health Care Quality Assessment
Source: J Med Internet Res. 2026 Mar 31;28:e81946. doi: 10.2196/81946 (PMC13037766; doi:10.2196/81946)
Supplement: Multimedia Appendix 2 [file jmir-v28-e81946-s002.docx]

**Multimedia Appendix 2.** Analysis of the available literature on PubMed.

We performed an analysis of the available literature on PubMed on 5^th^ January 2026; the summary of this research is reported in Table 5.

The research was performed via the following methodology:

- **First:** access to the PubMed portal: <https://pubmed.ncbi.nlm.nih.gov/>
- **Second,** in the portal, we introduce the following queries:
- **“Patient outcomes” AND: “Machine learning"**
- **“Patient outcomes” AND: “Graph Neural Networks"**
- **“Patient outcomes” AND “Digital Twins"**
- **Third**, the summary from the query has been visualized as a timeline on PubMed. The timeline has been downloaded from PubMed and stored as csv data. The extracted raw data as a CSFV data file are presented in Table S1.

Table S1 CSV data for different queries regarding patient outcomes and digital twins, graph neural networks and machine learning. A summary of these data is provided in Table 5.

| **Patient outcomes and Digital Twins** | **Patient outcomes and Graph Neural Networks** | **Patient outcomes and Machine Learning** |
| --- | --- | --- |
| Year,Count | Year,Count | Year,Count |
| 2025,161  2024,63  2023,20  2022,7  2021,7  2020,5  2019,2 | 2025,55  2024,39  2023,20  2022,16  2021,10  2020,7  2019,13 | 2025,7146  2024,4701  2023,3192  2022,2662  2021,2289  2020,1517  2019,899 |
