# Supplementary material for: From Knowledge Graphs to Digital Twins: Perspectives on Modeling Patient Outcomes for Health Care Quality Assessment
Source: J Med Internet Res. 2026 Mar 31;28:e81946. doi: 10.2196/81946 (PMC13037766; doi:10.2196/81946)
Supplement: Multimedia Appendix 3 [file jmir-v28-e81946-s003.docx]

Appendix 3

In Table A3-1, we present a short and essential literature overview of the application of graph models to model patient safety, procedure accuracy and procedure efficacy (following the taxonomy introduced in this article).

Table A3-1 List of relevant publications ordered by patient safety, procedure accuracy, and procedure efficacy.

| \| Quality categories \| Publication Details \| \| --- \| --- \| \| **Patient Safety** \| \| **Description - Publication** \| **Model Target** \| **Reference** \| \|  \| \| \| --- \| --- \| --- \| --- \| --- \| --- \| \| Prediction of Admission to ICU, where each node represents a patient with clinical data embeddings. The goal is to label a patient with either admission (1) or no admission (0) to the ICU (binary output). \| Prediction Hospital Readmission \| Recheteau et al. [1] \| \|  \| \| \| Hybrid model combining Long Short-Term Memory networks (LSTMs) for extracting temporal features and Graph Neural Networks (GNNs) for extracting patient neighborhood information patient to perform outcome predictions, like In-Hospital Mortality (IHM, i.e., Failure to Rescue) and Length of ICU Stay (LOS). \| Outcome Prediction: IHM, LOS \| Tong. et al. [2] \|  \| \| \| Model integrating image data and EHR in subnetworks. They have also developed a method to fuse outcomes from the different subnetworks, to analyze the probability of patient readmissions in cardiology. \| Probability Patient readmission \| Tang et al. [3] \|  \| \| \| Graph Neural Network for patient centered modeling with the integration of relevant clinical information to identify potential 30-day Hospital Readmissions. In this work, they provided diagnoses of acute myocardial infarction of anterolateral wall (ICD code: 410.0) and acute myocardial infarction of inferolateral wall (ICD code: 410.2), which are grouped under the general family diagnosis acute myocardial infarction (ICD code: 410). \| Potential 30-Day Hospital readmission \| Theodoropoulos et al. [4] \|  \| \| \| method combining GNNs and deep learning to predict Hospital Readmission from nonstructured data with a method that extracts deep representations of clinical notes using a feature aggregation unit on top of a state-of-the-art Natural Language Processing (NLP) technique - Bidirectional Encoder Representations from Transformers (BERT). By exploiting these deep representations, a patient network is built, and a Graph Neural Network (GNN) is trained to predict Hospital Readmissions. \| Prediction Hospital Readmission \| Golmaei et al. [5] \|  \| \| \| \| \|  \|  \| \| **Procedure accuracy** \| \| **Description - Publication** \| **Model Target** \| **Reference** \| \| --- \| --- \| --- \| \| Graph model representing patients grouped by similar diagnoses [6]. In this model, patients with similar gender, age and diagnoses (coded with ICDs) are linked. This methodology allows the representation of a cohort by means of a graph. Based on this structure the authors assumed that procedure predictions are a form of patient classification task. Naturally, this model is limited in regard to performing predictions for single patients as it is excluding metabolomic and genomic data. \| Procedure Prediction \| Diaz Ochoa & Mustafa [6] \| \| G-BERT (Graph – BERT method) for medical code representation and medication recommendation (i.e., procedure prediction)[7]. In this work, the authors implemented GNNs to represent the internal hierarchical structures of medical codes. In this investigation the authors integrate the GNN representation into a transformer-based visit encoder and pretrain it on EHR data from only a single visit. Pretrained visit representations are then fine-tuned for downstream predictive tasks on longitudinal EHRs from patients with multiple visits. \| Procedure Prediction \| Shang et al. [7] \| \| \|  \|  \| \| **Procedure efficacy** \| \| **Description - Publication** \| **Model Target** \| **Reference** \| \| --- \| --- \| --- \| \| Geometric Graph Neural Networks (GGNN) are a well-suited method to incorporate geometric features, associated with genomic data, into deep learning for enhanced predictive power and interpretability for multiomics data. Zhu et al. implemented this method for the evaluation of patients with myeloma as well as other cancers from the Cancer Atlas to perform a prognosis prediction and assist physicians in their decision-making process [8]. This implies the possibility of using such methods not only for patient monitoring but also as a basis for the evaluation of potential responses of patients to current or potential procedures. \| Prediction of patients with probability to develop Myeloma \| Zhu et al. [8] \| \| This study introduces a Graph-based Multimodal Late Fusion (GMLF) deep learning framework that integrates histopathological images and transcriptomic data to predict therapeutic response in muscle-invasive bladder cancer. By leveraging a knowledge graph built from known biological interactions, the model guides the representation of molecular data in a biologically meaningful way. The approach enables cross-modal learning, improves predictive efficacy, and provides interpretable insights into relevant biomarkers. The integration of domain knowledge into the graph structure enhances the model's ability to support personalized treatment decisions in oncology. \| Prediction of therapeutic response in muscle-invasive bladder cancer \| Bai et al. [9] \| \| Graph Neural Network (GNN) model to predict immunotherapy response in skin melanoma. By identifying key pathways and constructing a gene signature called response Score, the model achieved high predictive accuracy and revealed strong associations with prognosis, genetic alterations, and the tumor microenvironment. Among the 13 contributing genes, one was identified as a potential therapeutic target due to its negative correlation with immune activity and poor prognosis. The findings support the clinical utility of GNNs in guiding immunotherapy and identifying biomarkers in melanoma. \| Predict immunotherapy response in skin melanoma \| Ye et al. [10] \| \| |
| --- | --- | --- | --- | --- | --- | --- | --- | --- | --- | --- | --- | --- | --- | --- | --- | --- | --- | --- | --- | --- | --- | --- | --- | --- | --- | --- | --- | --- | --- | --- | --- | --- | --- | --- | --- | --- | --- | --- | --- | --- | --- | --- | --- | --- | --- | --- | --- | --- | --- | --- | --- | --- | --- | --- | --- | --- | --- | --- | --- | --- | --- | --- | --- | --- | --- | --- |

1. Rocheteau E, Tong C, Veličković P, Lane N, Liò P. Predicting Patient Outcomes with Graph Representation Learning. arXiv; 2021. doi: 10.48550/arXiv.2101.03940

2. Tong C, Rocheteau E, Veličković P, Lane N, Liò P. Predicting Patient Outcomes with Graph Representation Learning. In: Shaban-Nejad A, Michalowski M, Bianco S, editors. AI Dis Surveill Pandemic Intell Intell Dis Detect Action Cham: Springer International Publishing; 2022. p. 281–293. doi: 10.1007/978-3-030-93080-6_20ISBN:978-3-030-93080-6

3. Tang S, Tariq A, Dunnmon JA, Sharma U, Elugunti P, Rubin DL, Patel BN, Banerjee I. Predicting 30-day all-cause hospital readmission using multimodal spatiotemporal graph neural networks. IEEE J Biomed Health Inform 2023 Jan 13;PP. PMID:37018684

4. Theodoropoulos C, Mulligan N, Stappenbeck T, Bettencourt-Silva J. Representation Learning for Person or Entity-Centric Knowledge Graphs: An Application in Healthcare. Proc 12th Knowl Capture Conf 2023 New York, NY, USA: Association for Computing Machinery; 2023. p. 225–233. doi: 10.1145/3587259.3627545

5. Golmaei SN, Luo X. DeepNote-GNN: predicting hospital readmission using clinical notes and patient network. Proc 12th ACM Conf Bioinforma Comput Biol Health Inform New York, NY, USA: Association for Computing Machinery; 2021. p. 1–9. doi: 10.1145/3459930.3469547

6. Diaz Ochoa JG, Mustafa FE. Graph neural network modelling as a potentially effective method for predicting and analyzing procedures based on patients’ diagnoses. Artif Intell Med 2022 Sept 1;131:102359. doi: 10.1016/j.artmed.2022.102359

7. Shang J, Ma T, Xiao C, Sun J. Pre-training of graph augmented transformers for medication recommendation: 28th International Joint Conference on Artificial Intelligence, IJCAI 2019. Kraus S, editor. Proc 28th Int Jt Conf Artif Intell IJCAI 2019 International Joint Conferences on Artificial Intelligence; 2019;5953–5959. doi: 10.24963/ijcai.2019/825

8. Zhu J, Oh JH, Simhal AK, Elkin R, Norton L, Deasy JO, Tannenbaum A. Geometric graph neural networks on multi-omics data to predict cancer survival outcomes. Comput Biol Med 2023 Sept;163:107117. PMID:37329617

9. Bai Z, Osman M, Brendel M, Tangen CM, Flaig TW, Thompson IM, Plets M, Scott Lucia M, Theodorescu D, Gustafson D, Daneshmand S, Meeks JJ, Choi W, Dinney CPN, Elemento O, Lerner SP, McConkey DJ, Faltas BM, Wang F. Predicting response to neoadjuvant chemotherapy in muscle-invasive bladder cancer via interpretable multimodal deep learning. Npj Digit Med Nature Publishing Group; 2025 Mar 22;8(1):174. doi: 10.1038/s41746-025-01560-y

10. Ye M, Ren S, Luo H, Wu X, Lian H, Cai X, Ji Y. Integration of graph neural networks and transcriptomics analysis identify key pathways and gene signature for immunotherapy response and prognosis of skin melanoma. BMC Cancer BioMed Central; 2025 Dec;25(1):1–18. doi: 10.1186/s12885-025-13611-4
