# Supplementary material for: From Knowledge Graphs to Digital Twins: Perspectives on Modeling Patient Outcomes for Health Care Quality Assessment
Source: J Med Internet Res. 2026 Mar 31;28:e81946. doi: 10.2196/81946 (PMC13037766; doi:10.2196/81946)
Supplement: Multimedia Appendix 4 [file jmir-v28-e81946-s004.docx]

**Multimedia Appendix 4.** Lifecycle of the data and models in relation to clinical guidelines.

All the methods described in this article belong within a lifecycle, as shown in Figure S1.

*
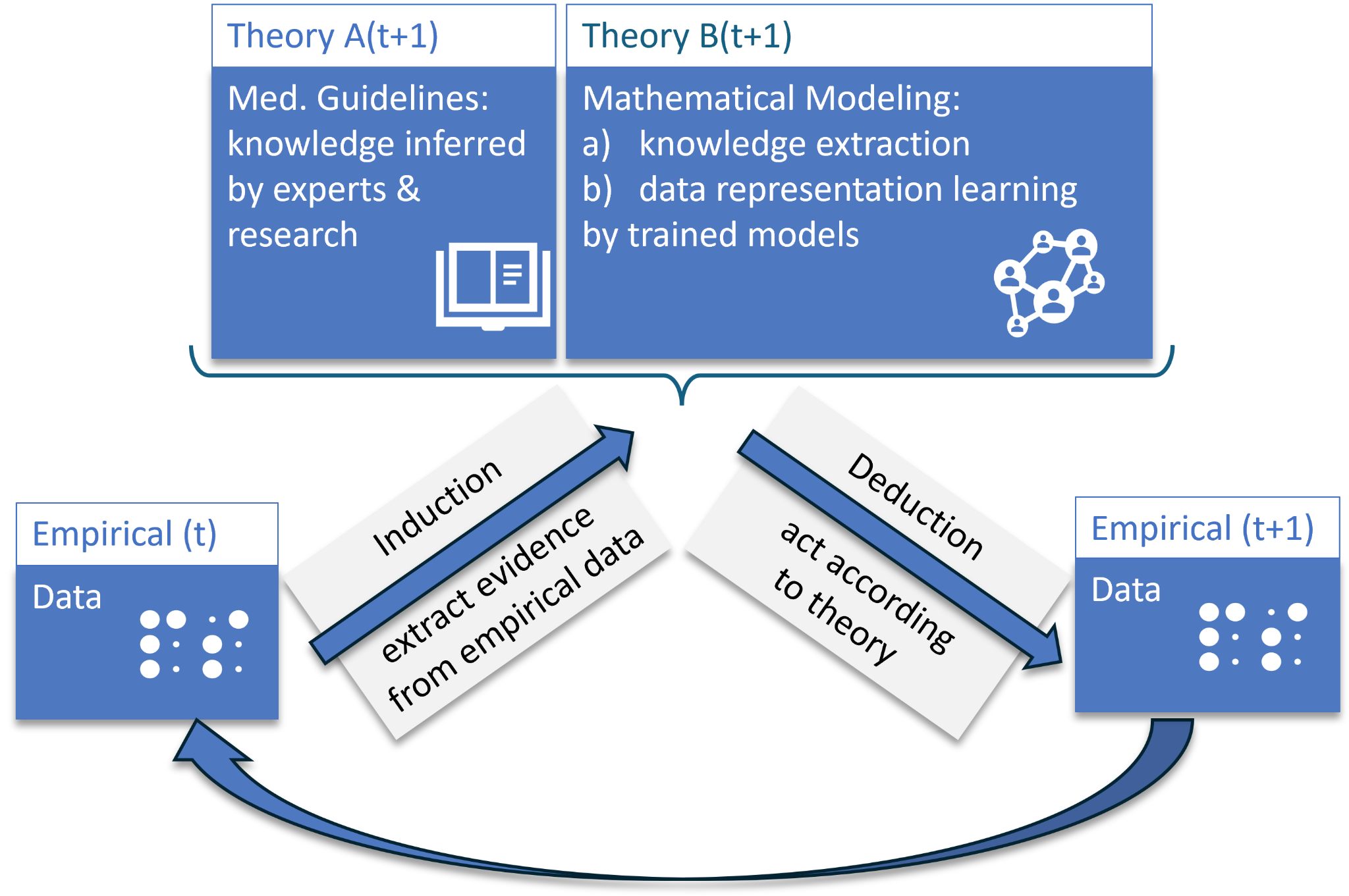
*

Figure S1 **Lifecycle of the data and models in relation to the clinical guidelines.** Concept induction, as the extraction of evidence from empirical data (t) and deduction, is the generation of new empirical data (t+1) by action according to a given general theory. The theory is, on the one hand, given by the medical guidelines – Theory A(t+1) and, on the other hand, by the mathematical models trained on the given empirical data – Theory B(t+1).

We understand mathematical modeling as a subsection of medical guidelines. We visualize the role of mathematical modeling in evidence generation and in improving healthcare. Furthermore, from time to time, empirical observations stored as data are evaluated to develop and adapt improvements to medical guidelines implemented by health professionals.
